# Which features of subjective cognitive decline are related to amyloid pathology? Findings from the DELCODE study

**DOI:** 10.1186/s13195-019-0515-y

**Published:** 2019-07-31

**Authors:** Lisa Miebach, Steffen Wolfsgruber, Alexandra Polcher, Oliver Peters, Felix Menne, Katja Luther, Enise Incesoy, Josef Priller, Eike Spruth, Slawek Altenstein, Katharina Buerger, Cihan Catak, Daniel Janowitz, Robert Perneczky, Julia Utecht, Christoph Laske, Martina Buchmann, Anja Schneider, Klaus Fliessbach, Pascal Kalbhen, Michael T. Heneka, Frederic Brosseron, Annika Spottke, Nina Roy, Stefan J. Teipel, Ingo Kilimann, Jens Wiltfang, Claudia Bartels, Emrah Düzel, Laura Dobisch, Coraline Metzger, Dix Meiberth, Alfredo Ramirez, Frank Jessen, Michael Wagner

**Affiliations:** 1 German Center for Neurodegenerative Diseases/Clinical Research, Deutsches Zentrum für Neurodegenerative Erkrankungen e.V. (DZNE), Zentrum für klinische Forschung/AG Neuropsychologie, Sigmund-Freud-Str. 27, 53127 Bonn, Germany; 2 Department for Neurodegenerative Diseases and Geriatric Psychiatry, University Hospital Bonn, Sigmund-Freud-Str. 27, 53127 Bonn, Germany; 3 Department of Psychiatry, Medical Faculty, University of Cologne, Cologne, Germany; 4 Institute of Psychiatry and Psychotherapy, Charité – Universitätsmedizin Berlin, Berlin, Germany; 5 German Center for Neurodegenerative Diseases (DZNE), Berlin, Germany; 6 Department of Psychiatry and Psychotherapy, Charité, Berlin, Germany; 7 Institute for Stroke and Dementia Research (ISD), LMU Munich, Munich, Germany; 8 German Center for Neurodegenerative Diseases (DZNE), Munich, Germany; 9 Ludwig-Maximilians-Universität München, Munich, Germany; 10 German Center for Neurodegenerative Diseases (DZNE), Tübingen, Germany; 11 Hertie-Institute for Clinical Brain Research, Tübingen, Germany; 12 Department of Neurology, University of Bonn, Bonn, Germany; 13 Department of Psychosomatic Medicine, University of Medicine, Rostock, Germany; 14 German Center for Neurodegenerative Diseases (DZNE), Rostock, Germany; 15 German Center for Neurodegenerative Diseases (DZNE), Goettingen, Germany; 16 Department of Psychiatry and Psychotherapy, University of Göttingen, Göttingen, Germany; 17 Institute of Cognitive Neurology and Dementia Research (IKND), Otto-von-Guericke University, Magdeburg, Germany; 18 German Center for Neurodegenerative Diseases (DZNE), Magdeburg, Germany; 19 Department of Psychiatry and Psychotherapy, Otto-von-Guericke University, Magdeburg, Germany; 20 Munich Cluster for Systems Neurology (SyNergy) Munich, Munich, Germany; 21 Neuroepidemiology and Ageing Research Unit, School of Public Health, Imperial College London, London, UK

**Keywords:** Preclinical Alzheimer’s disease (AD), Subjective cognitive decline (SCD), Cerebrospinal fluid (CSF), Aß42, Preclinical AD, CSF biomarkers

## Abstract

**Background:**

Subjective cognitive decline (SCD) has been proposed as a pre-MCI at-risk condition of Alzheimer’s disease (AD). Current research is focusing on a refined assessment of specific SCD features associated with increased risk for AD, as proposed in the SCD-plus criteria. We developed a structured interview (SCD-I) for the assessment of these features and tested their relationship with AD biomarkers.

**Methods:**

We analyzed data of 205 cognitively normal participants of the DELCODE study (mean age = 68.9 years; 52% female) with available CSF AD biomarkers (Aß-42, p-Tau181, Aß-42/Tau ratio, total Tau). For each of five cognitive domains (including memory, language, attention, planning, others), a study physician asked participants about the following SCD-plus features: the presence of subjective decline, associated worries, onset of SCD, feeling of worse performance than others of the same age group, and informant confirmation. We compared AD biomarkers of subjects endorsing each of these questions with those who did not, controlling for age. SCD was also quantified by two summary scores: the number of fulfilled SCD-plus features, and the number of domains with experienced decline. Covariate-adjusted linear regression analyses were used to test whether these SCD scores predicted abnormality in AD biomarkers.

**Results:**

Lower Aß-42 levels were associated with a reported decline in memory and language abilities, and with the following SCD-plus features: *onset of subjective decline within 5 years*, *confirmation of cognitive decline by an informant*, and *decline-related worries*. Furthermore, both quantitative SCD scores were associated with lower Aß42 and lower Aß42/Tau ratio, but not with total Tau or p-Tau181.

**Conclusions:**

Findings support the usefulness of a criterion-based interview approach to assess and quantify SCD in the context of AD and validate the current SCD-plus features as predictors of AD pathology. While some features seem to be more closely associated with AD biomarkers than others, aggregated scores over several SCD-plus features or SCD domains may be the best predictors of AD pathology.

**Electronic supplementary material:**

The online version of this article (10.1186/s13195-019-0515-y) contains supplementary material, which is available to authorized users.

## Background

Subjective cognitive decline (SCD), the subjective experience of worsening cognitive performance among cognitively normal older individuals, can indicate an at-risk stage of Alzheimer’s disease (AD) [1, 2]. Several studies, using a variety of assessments, found SCD to predict objective cognitive decline [3, 4], incident mild cognitive impairment (MCI) [5] and incident AD dementia [5, 6].

Furthermore, in several cross-sectional studies, cognitive complaints were found to correlate with biomarkers of early AD pathology such as amyloid-ß (Aß). For example, Amariglio and colleagues [7] found an association of Aß deposition in the brain and a memory complaint composite score in cognitively normal older adults. Higher baseline memory complaint scores in participants screened positive for Aß were also found to predict faster cognitive decline [8].

These and other studies have established that some form of SCD can be a clinical indicator of early AD (stage 2, according to the NIA-AA research framework [9]).

Based on the evidence accrued until 2014, a group of researchers forming the SCD-Initiative proposed the “SCD-plus criteria” as an enrichment strategy for the likelihood of preclinical AD in individuals with SCD [9], comprising (a) *Subjective decline in memory rather than other domains*, (b) *onset of SCD within the last 5 years*, (c) *age of onset ≥ 60 years*, (d) *particular concerns associated with SCD*, (e) *the feeling of worse performance than others of the same age group*, (f) *confirmation of perceived cognitive decline by an informant*, and (g) the *presence of the APOE e4 genotype*.

These criteria were not meant to be final but were considered to be in need of further refinement and validation in research studies. For example, recent studies suggest that consistency of complaints over time may be another feature associated with the presence of AD risk [6].

Current assessments differ widely regarding administration (interview with a physician versus questionnaire), content, number of items, and scaling, leading to a large variety of methods [10, 11]. While some SCD studies used single questionnaire items [12] or items from different SCD questionnaires [13–15], others were using one out of many questionnaires [7, 11] or even composites derived from several questionnaires (e.g., [16]). Psychometric analyses are ongoing to extract from existing data those single SCD questions or features contributing most to the prediction of AD [17].

One potential limitation of most current SCD assessments is that they only refer to memory [11]. Subjective memory concerns are highly prevalent in older adults (e.g., around 53% in a large population-based sample [18]) and may therefore be highly sensitive but of insufficient specificity regarding the detection of preclinical AD. Thus, current research suggests involving additional cognitive domains in SCD assessment [2], e.g., subjective complaints in executive function which have also been associated with Aß deposition in cognitively normal individuals [7]. Irrespective of the cognitive domains, studies have also highlighted specific features of SCD which are associated with AD biomarkers, objective cognitive decline, or incident MCI. Perrotin and colleagues [19] for example found an association between the comparison of memory function to peers with Aß deposition using Pittsburgh compound B positron emission tomography (PiB-PET) imaging.

Another feature replicated in several studies is the presence of worries associated with the subjective worsening in function.

A recent study on the validity of SCD-plus criteria in cognitively unimpaired patients of the Amsterdam memory clinic [20] could not find significant relationships between amyloid biomarkers and any of the examined subjective cognition features (“memory specific decline”, “onset of complaints within 5 years”, “worse performance than others of the same age”, and “informant reports decline”). Amyloid was only predicted by higher age (> 60) and ApoE4 in this study, in line with established knowledge [21]. Apart from sample size limitations in this study, the apparent insensitivity of the subjective cognition features in the SCD-plus criteria could have been due to the relatively young age of the memory clinic subjects (64 years on average) and to the measurement of SCD with two different questionnaires which were not designed to fully capture the SCD-plus criteria.

Importantly, there is no straightforward, interview-based assessment of SCD criteria, and a single validation study of SCD-plus features is still missing. In clinical settings, structured interviews offer an advantage over questionnaires as they rely on personal contact to the patient, thus improving acceptance, and may allow an informed clinical rating of participants’ complaints according to diagnostic categories. They are an established strategy for reducing information variance [22, 23].

In the present study, we aimed to provide further validation of the SCD-plus features while also testing the usefulness of an interview-based assessment for AD-related SCD. We developed a new, semi-structured interview for detailed SCD assessment (SCD-I) which includes assessment of perceived decline in different cognitive domains as well as the SCD-plus criteria mentioned above. We examined cognitive complaints as measured by the SCD-I in a sample of cognitively normal older adults and tested for associations of individual SCD features as well as composite scores derived from the interview with biomarkers of AD pathology, respectively.

## Methods

### Study design

The DZNE-Longitudinal Cognitive Impairment and Dementia Study (DELCODE) is an observational longitudinal memory clinic-based multicenter study in Germany with the aim to improve characterization of the early, preclinical stage of AD with a focus on SCD patients. The study protocol was approved by Institutional Review Boards of all participating study centers of the DZNE [24]. All patients provided written informed consent.

### Participants

We included 205 participants (age M = 68.9; SD = 5.4) from an interim data release of the DELCODE study. Here, we analyzed only data from cognitively normal individuals. These included healthy controls (HC, *n* = 76) who all had denied any worrisome subjective cognitive impairment during an initial telephone screening for study eligibility, first-degree relatives of patients with AD dementia (AD relatives, *n* = 24), and memory clinic patients with unimpaired test performance but with a report of worrisome subjective cognitive decline at the initial screening (SCD patients, *n* = 105).

The diagnostic criteria for group definition and the study protocol have been described in detail previously [24]. The HC and the AD relatives group were both recruited via local newspaper advertisement and conducted a telephone interview to screen for suitability. The SCD patient group was recruited via the memory clinics of all participating DELCODE sites. These individuals sought diagnostic evaluation of subjectively experienced a decline in cognitive functioning. It was required that they expressed concerns to the physician of the memory clinic regarding their self-perceived cognitive decline while their test performance was above − 1.5 SD of age-, sex-, and education-adjusted normal performance on all subtests of the CERAD neuropsychological assessment battery. Subsequent to these different screening procedures, subjects in all groups were enrolled into the DELCODE study and underwent a uniform baseline assessment including the semi-structured SCD interview described below. 

### Subjective cognitive decline interview (SCD-I) and scoring procedures

The SCD-I allows assessment of subjective cognitive decline in five different cognitive domains *(memory*, *language*, *planning*, *attention*, *any other cognitive decline)* and comprises all five SCD-plus features which refer to subjective experience [2]. All interviews were administered face to face by trained study physicians and lasted approximately 5 min. The interview consists of 3 parts including an open question at the beginning as well as a structured part for the participant and the informant. In this study, we are only focusing on the structured part. The whole interview procedure is shown in Additional file 1. For each domain, the physician asked the patient if he/she had noticed any worsening in function (e.g., “do you feel like your memory has become worse”)*.* If the participant answered this question with yes, the physician added more in-depth questions about the domain to assess the presence/absence of SCD-plus features, i.e., specific questions about associated worries (“Does this worry you?”), onset (“How long ago did you start to notice the decline?”), and the performance in comparison to peers (“Compared to other people of your age, would you say that your performance is worse?”). Furthermore, participants were asked whether they had talked to a physician about their subjective cognitive decline (this information was not analyzed in the present study as by design all SCD subjects had been referred to a memory clinic). In addition, a modified SCD-I was administered to a study partner (usually a relative or spouse) of all participants, asking for an *observed* decline in any of the same five domains. Study partners were not asked the in-depth SCD-plus questions but were also asked about whether they had observed any behavioral changes in the participant (this was not analyzed in the present study).

The quantification of response data allows derivation of the total number of domains with a reported decline as well as the total number of fulfilled SCD-plus features. This scoring was executed as follows:

*Number of fulfilled SCD-plus features*: Reported as number of fulfilled SCD-plus features ranging from 0 to 5 (decline in memory, onset within the last 5 years, worries associated with a decline in a cognitive domain, feeling of worse performance than others of the same age group, confirmation of perceived cognitive decline by an informant).

*Number of reported SCD domains*: Sum of the number of cognitive domains (memory, language, planning, attention, others) in which the participant endorses a worsening in function (maximum score = 5).

### Neuropsychological and clinical assessment

The DELCODE test battery included an extensive neuropsychological and clinical assessment which covers tests for global cognitive function and different cognitive domains (described in detail previously [24]) as well as a structured medical history and a standardized physical examination [24]. Here, we focus on the assessment relevant to the present study. The Mini-Mental State Examination (MMSE) is used to describe the global cognitive function in all subgroups, and the 15-item short form of the Geriatric Depression Scale (GDS) to measure and control for depressive symptomatology. In a previous memory clinic study [25], we had found that questions on SCD (different from those in the SCD-I) were associated with CSF AD biomarkers, and this was still true after controlling for delayed recall memory performance (an established, strong predictor of CSF AD biomarkers [26]). We analyzed the present data in the same manner with the ADAS delayed recall as covariate.

### CSF AD biomarker measures

CSF samples were collected according to previously described standard operating procedure [24] by using commercially available kits according to vendor specifications (V-PLEX Aβ Peptide Panel 1 (6E10) Kit (Intra Plate variance of 3.0 and inter plate variance of 8.8), K15200E and V-PLEX Human Total Tau Kit (intra plate variance of 4.5 and an inter plate variance of 17.1), K151LAE (Mesoscale Diagnostics LLC, Rockville, USA), and Innotest Phospho-Tau (181P) (intra plate variance of 1.7 and inter plate variance of 11.4), 81581, Fujirebio Germany GmbH, Hannover, Germany).

We used the continuous variables of Aß-42 level, the p-tau-181, and the total Tau level as outcomes. In addition, we calculated a CSF amyloid/tau ratio score (Aβ42/(240 + 1.18 × tau) which has been established as a specific marker for AD [27]. We decided to use continuous biomarker values (rather than categorical variables based on cutoffs) in order to explore associations of SCD within the complete spectrum of AD pathological change, especially Aβ accumulation, in cognitively normal individuals, i.e. without loss of information due to dichotomization. This is supported by recent study results, which showed that Aβ accumulation, in cognitively normal older participants still classified as Aß-negative, was associated with longitudinal changes in memory function [28].

### Statistical analysis

All analyses were performed using SPSS Version 23.0 (IBM) for Windows. For descriptive statistics, we used *x*^*2*^ test for categorical and analyses of variance for continuous variables as well as post hoc *t* tests or chi-square tests for single contrasts. Group differences in CSF level were reported as age-adjusted results based on ANCOVA. Linear regression models were used to examine the relationship between different SCD score and the CSF biomarker outcome variables described above. We performed separate analyses for the number of fulfilled SCD-plus features as well as for the number of reported SCD domains. In step 1, we entered one of the SCD scores as a single predictor. In a second step, we adjusted for age, sex, and education. In order to gauge the “added benefit” of SCD questions over and above memory testing, we controlled for objective memory performance by using the world list delayed recall score as a covariate. All cases with missing data in any variables were excluded. Since we included just participants with CSF biomarkers, we tested whether our sample differed significantly from cognitively normal DELCODE participants without biomarkers (*n* = 291). The samples did not differ in terms of age (*t*(493) = − 1.84; *p* = .067), sex (*X*^2^ = .441; *p* = .507), and education (*t*(491) = − .304; *p* = .761) and neither regarding the number of fulfilled SCD plus features (*t*(493) = − .288; *p* = .774) or the number of reported SCD domains (*t*(493) = .969; *p* = .333).

## Results

### Sample descriptive statistics and group differences in demographic, clinical, cognitive, and biomarker data

The 205 included participants (of whom 107 (52.2%) were female) had a mean age of 69 years (*SD* = 5.4) and mean education of 14.7 years (*SD* = 2.95). Demographic, neuropsychological, and clinical characteristics of the sample as well as detailed group differences are shown in Table 1. HC, AD relatives, and SCD patients did not differ with regard to sex, education, MMSE, and word list delayed recall score, although we note SCD patients and AD relatives had slightly worse memory performance. AD relatives were younger than SCD patients and HC, while SCD patients had slightly higher scores in the GDS, which however where in the normal range (GDS < 6) in most cases (97.3%).

**Table 1:** Sample characteristics and group differences in CSF biomarkers

*N* = 205	Total Sample	HC (*n* = 76)	HC vs. SCD^b^	SCD (*n* = 105)	SCD vs. Rel^b^	Relatives of AD (*n* = 24)	HC vs. Rel^b^	*F* value/chi^2^ value^c^
Age (in years, mean, SD)	68.9 (5.4)	68.3 (4.9)	*	70.4 (5.5)	***	64.5 (3.7)	**	14.2***, *p* < 0.001
Sex (female; *n*, %)	107 (52.2)	42 (55.3)		48 (45.7)	*	17 (70.8)		5.4, *p* = .07
Education (in years, mean, SD)	14.7 (2.9)	14.6 (2.8)		15.0 (3.1)		13.6 (2.5)		2.1, *p* = .12
MMSE (mean, SD)	29.26 (0.95)	29.4 (0.9)		29.2 (0.9)		28.9 (1.2)		2.38, *p* = .09
Word list recall (mean, SD)	7.5 (1.7)	7.9 (1.6)		7.3 (1.7)		7.1 (2.2)		2.97, *p* = .053
GDS (mean, SD)	1.27 (1.6)	0.7 (1.3)	***	1.8 (1.8)	**	1.0 (1.5)		13.05***, *p* < 0.001
APOE genotype								
APOE4 genotype of all APOE (*n*, %)	53 (25.9)	15 (21.4)		31 (33.7)		7 (43.8)		4.50, *p* = 0.105
CSF biomarkers^a^								
Aß42 (pg/ml; mean, SD)	768.55 (313.89)	851.80 (301.55)	**	708.64 (316.88)		767.00 (289.93)		4.23*, *p* < .05
Aß42/Tau ratio (mean, SD)	1.15 (0.48)	1.28 (0.47)	**	1.06 (0.49)		1.14 (0.40)		4.02*, *p* < .05
Total Tau (mean, SD)	393.46 (175.63)	384.73 (165.199)		404.506 (195.333)		372.799 (103.895)		0.081, *p* = .923
p-Tau-181 (pg/ml; mean, SD)	51.00 (20.82)	51.22 (18.51)		51.73 (23.94)		46.81 (12.32)		0.94, *p* = .940

Bonferroni adjusted *p* values; *p* value = .05, two-tailed sign

*SD* standard deviation, *MMSE* Mini-Mental State Examination, *GDS* Geriatric Depression Scale, *HC* Healthy Controls

*Significant results on the *α* < .05 level

**Significant results on the *α* < .01 level

***Significant results on the *α* < .001 level

^a^Tests of CSF biomarkers are adjusted for age

^b^Post hoc *t* tests for continuous and chi^2^ tests for categorical variables (sex and APOE4)

^c^*F* values were presented for continuous variables, chi^2^ values for categorical variables (sex and APOE4)

The three groups differed significantly in the CSF-Aß42 level and the Aß42/Tau ratio (see Table 1) after adjusting for age. The SCD group had a significantly lower Aß-42 concentration and a significantly lower Aß42/tau ratio relative to the HC group. There was no significant group difference in p-tau-181 level and in t-tau level (see Table 1).

### Prevalence and group differences of SCD-plus features and SCD domains

**Fig. 1:**
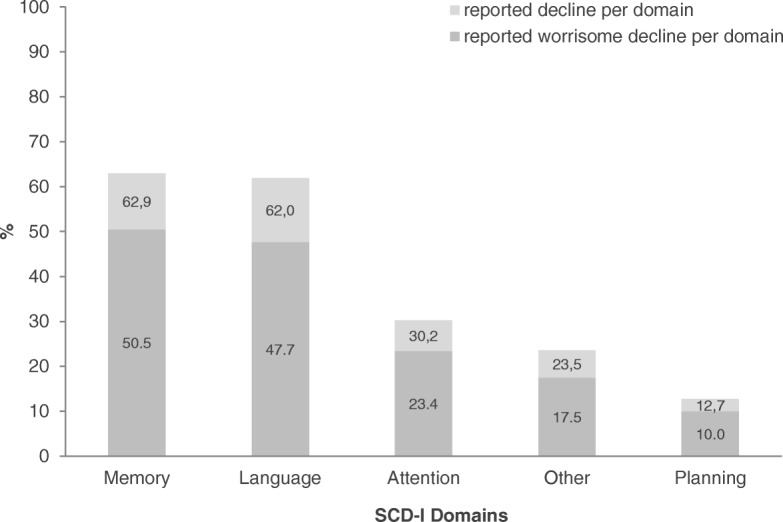
Frequency of domains reported with an experienced decline and associated worries, respectively

An overview of the prevalence and the group differences in SCD-plus features is given in Table 2. Reported domains in the total sample are shown in Fig. 1. Out of the 205 individuals, 76.1% reported a cognitive decline in at least one domain, and among those experiencing a decline, 72% also endorsed worries associated with the decline. Most complaints were reported in the memory (*n* = 129; 62.9%) and language domain (*n* = 127; 62%).

**Table 2:** Prevalence and group differences in SCD-plus features and SCD-I domains

*N* = 205	Sample					Chi^2^*/F* value; *p* value
		Total Sample	HC (*n* = 76)	SCD (*n* = 105)	Relatives of AD (*n* = 24)	
SCD-plus features						
Decline in memory	Yes, *n* (%)	129 (62.9)	20 (26.3)	98 (93.3)	11 (45.8)	88.285***, *p* < .001
	No, *n* (%)	76 (37.1)	56 (73.7)	7 (6.7)	13 (54.2)	
Onset of SCD in any domain within the last 5 years	Yes, *n* (%)	120 (58.5)	24 (44.5)	85 (81.0)	11 (45.8)	46.088***, *p* < .001
	No, *n* (%)	85 (41.5)	52 (68.4)	20 (19.0)	13 (54.2)	
Particular concerns/worries in any domain	Yes, *n* (%)	113 (55.1)	11 (14.5)	95 (90.5)	7 (29.2)	110.351***, *p* < .001
	No, *n* (%)	92 (44.9)	65 (85.5)	10 (9.5)	17 (70.8)	
The feeling of worse performance than others in any domain	Yes, *n* (%)	35 (17.1)	1 (1.3)	31 (29.5)	3 (12.5)	25.179***, *p* < .001
	No, *n* (%)	170 (82.9)	75 (98.7)	74 (70.5)	21 (87.5)	
Confirmation of decline in any domain by an informant^a^	Yes, *n* (%)	81 (39.5)	15 (39.47)	59 (57.84)	7 (43.75)	25.731***, *p* < .001
	No, *n* (%)	124 (60.5)	61 (60.53)	46 (42.16)	17 (56.25)	
Number of fulfilled SCD-plus features	*M (SD)*	2.33 (1.73)	0.9 (1.27)	3.5 (1.08)	1.63 (1.71)	99.81***, *p* < .001
SCD-I domains						
Decline in memory	Yes, *n* (%)	129 (62.9)	20 (26.3)	98 (93.3)	11 (45.8)	88.285***, *p* < .001
	No, *n* (%)	76 (37.1)	56 (73.7)	7 (6.7)	13 (54.2)	
Decline in language	Yes, *n* (%)	127 (62.0)	28 (36.8)	87 (82.9)	12 (50)	41.251***, *p* < .001
	No, *n* (%)	78 (38.0)	48 (63.2)	18 (17.1)	12 (50)	
Decline in attention	Yes, *n* (%)	62 (30.2)	6 (7.9)	49 (46.7)	7 (29.2)	31.430***, *p* < .001
	No, *n* (%)	143 (69.8)	70 (92.1)	56 (53.3)	17 (70.8)	
Decline in planning	Yes, *n* (%)	20 (9.8)	2 (2.6)	22 (21.0)	2 (8.3)	13.827***, *p* < .001
	No, *n* (%)	6 (2.9)	74 (97.4)	83 (79.0)	22 (91.7)	
Decline in other	Yes, *n* (%)	48 (23.5)	5 (6.6)	39 (37.1)	4 (16.7)	24.045***, *p* < .001
	No, *n* (%)	156 (76.1)	71 (93.4)	65 (61.9)	20 (83.3)	
Number of reported SCD domains	*M (SD)*	1.91 (1.47)	0.80 (0.98)	2.81 (1.17)	1.50 (1.44)	70.17***, *p* < .001

*p < .05

**p < .01

***p < .001

^a^This refers to participants reporting a decline in at least one domain including a total of *n* = 156 (76.1%) individuals (HC: *n* = 38 (50%); SCD: *n* = 102 (97.1%); AD relatives *n* = 16 (66.7%))

As expected, due to the inclusion criteria, the three participant groups differed in the endorsement of decline in SCD domains and SCD-plus features (see Table 2). Unsurprisingly, most (93.3%) SCD patients reported a decline in memory, but a sizeable proportion of the other participants also did, although less frequently (HC 26.3%; comparison with SCD, *X*^2^ = 87.26; *p* < .001; AD relatives 45.8%; comparison with SCD *X*^2^ = 33.65; *p* < .001). The same pattern was observed for experienced decline in language abilities (SCD = 82.9%, HC = 36.8%, pairwise comparison *X*^2^ = 40.29; *p* < .001; AD relatives = 50%, pairwise comparison to SCD *X*^2^ = 11.82; *p* < .001).

The *number of domains with* a reported decline differed significantly across the groups (*F*(2,202) = 70.17, *p* < .001). On average, the SCD group mentioned a decline in two domains, while the number of impaired domains was 0.8 in the healthy control group (*p*_(bonf. adj.)_ ≤ .001) and 1.5 in the AD relatives’ group (*p*_(bonf. adj.)_ ≤ .001).

Group differences also emerged regarding the reported onset of decline in participants reporting any such decline. Around 80% of SCD patients reported an onset of cognitive worsening (in any domain) within the last 5 years. This was significantly more often than in the HC group (44.5%; *X*^2^ = 44.87; *p* < .001) and in the AD relatives (45.8%; *X*^2^ = 12.66; *p* < .001), who more often reported a more distant onset of decline. There was no significant difference between HC and AD relatives (*X*^2^ = 1.63; *p* = .20).

The feeling of worse performance than others (in any domain) was also most frequently reported in the SCD group (29.5%) compared to healthy controls (1.3%) (*X*^2^ = 24.10; *p* < .001) and relatives of AD patients (12.5%; *X*^2^ = 2.92; *p* = .088). AD relatives also reported this slightly more often than HC (*X*^2^ = 5.94; *p* = .042).

Interestingly, although all HC participants had negated a worrisome cognitive decline during the initial telephone screening, a worrisome decline (in any domain) was reported by 14.5% of HC during the physician-led personal interview. In AD-relatives, where the absence of worrisome cognitive decline was not an exclusion criterion, the prevalence was 29.2%. As expected because of the inclusion criteria, SCD patients reported concerns much more frequently (90.5%) compared to both at-risk groups (SCD vs. HC: *X*^2^ = 104.58; *p* < .001; SCD vs. AD relatives: *X*^2^ = 44.37; *p* < .001), which did not differ from each other.

The informant also reported (i.e., confirmed) a decline in at least one domain for the majority (57.8%) of the SCD patients who reported a decline by themselves in at least one domain, while such a confirmation occurred less often in the HC group (39.5%, *X*^2^ = 24.24; *p* < .001) and in AD relatives (43.8%, *X*^2^ = 5.710; *p* < .05).

The number of fulfilled SCD-plus features differed highly significantly between the three groups (*F*(2,202) = 99.807; *p* < .001). Participants in the SCD group fulfilled more SCD-plus features (*M* = 3.5) than participants in the HC group (*M* = 0.93, *p*_(bonf. adj.)_ ≤ .001) and in the AD relatives (M = 1.63, *p*_(bonf. adj.)_ ≤ .001), which also differed from each other (*p*_(bonf. adj.)_ ≤ .05).

### Relationship between AD biomarkers and SCD plus features and SCD domains

In the combined sample of all three groups, lower age-adjusted CSF-Aß-42 levels were found in those fulfilling the SCD-plus features *of a decline in memory* (*F*(1,202) = 7.65, *p* < .01, ${\eta}_p^2$ = .036), *onset within the last 5 years* (*F*(1,202) = 6.07, *p* < .05, ${\eta}_p^2$ = .029), and the *confirmation by an informant (F*(1,202) = 4.19, *p* < .05, ${\eta}_p^2$ = .032, Table 3). The association of lower CSF-Aß-42 with *worries in any domain* approached significance *(F*(1,202) = 3.68, *p* = .056, ${\eta}_p^2$ = .018).

**Table 3:** Associations between endorsement of SCD-plus features and SCD-I domains with CSF-Aß-42 level

*N* = 205	CSF-Aß-42 level (pg/ml)						*p* ^*a*^
			*M*	(SD)	*F*	${\eta}_p^2$	
SCD-plus features							
Decline in memory	*Yes*	*n* = 129	720	− 316	7.65**	.036	.006
	*No*	*n* = 76	849	− 293			
Onset of SCD within the last 5 years	*Yes*	*n* = 120	722	− 312	6.07*	.029	.015
	*No*	*n* = 85	833	− 306			
Particular concerns/worries	*Yes*	*n* = 113	727	− 309	3.68	.018	.056
	*No*	*n* = 92	819	− 313			
The feeling of worse performance than others	*Yes*	*n* = 35	695	− 308	2.488	.012	.116
	*No*	*n* = 170	783	− 313			
Confirmation by an informant	*Yes*	*n* = 81	695	− 315	4.19*	.032	.017
	*No*	*n* = 124	816	− 304			
SCD-I domains							
Decline in memory	*Yes*	*n* = 129	720	− 316	7.65**	.036	.006
	*No*	*n* = 76	849	− 293			
Decline in language	*Yes*	*n* = 127	727	− 312	5.18*	.025	.024
	*No*	*n* = 78	835	− 306			
Decline in attention	*Yes*	*n* = 62	738	− 349	.751	.004	.387
	*No*	*n* = 143	781	− 297			
Decline in planning	*Yes*	*n* = 26	704	− 326	1.049	.005	.307
	*No*	*n* = 179	777	− 312			
Decline in other	*Yes*	*n* = 48	716	− 302	1.65	.008	.201
	*No*	*n* = 156	780	− 313			

*M* mean, *SD* standard deviation

*p < .05

**p < .01

***p < .001

^*a*^Adjusted for age for the SCD-I domains and for age and education for the SCD-plus features, ${\eta}_p^2$ > .01 = small effect; ${\eta}_p^2$ > .06 = average effect; ${\eta}_p^2$ > .14 = large effect (according to Cohen 1988)

**Fig. 2:**
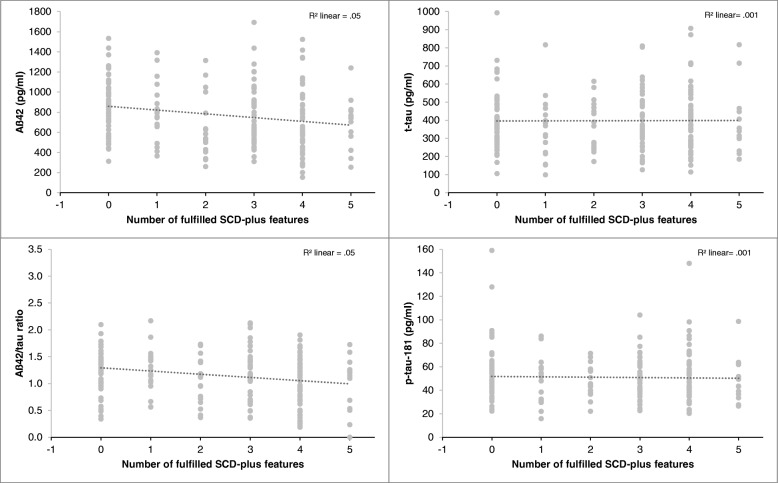
Relationship between CSF biomarkers and the number of fulfilled SCD-plus features in cognitively normal individuals (*n* = 205)

Hierarchical linear regression analysis showed that the number of fulfilled SCD-plus features was a significant predictor of a reduced (more pathological) CSF-Aß-42 level (*ß* = − .225, *p* < .005) (Fig. 2) and of a reduced (more pathological) CSF Aß-42/tau-ratio (*ß* = − .189, *p* < .01) independent of age, sex, and education. In contrast, the relationship between the number of fulfilled SCD-plus features and CSF total Tau (*ß* = − .055, *p* > .05) and p-tau-181 (*ß* = − .077, *p* > .05) was not significant.

Using objective memory performance (word list delayed recall) as an additional covariate to control for subtle group deficits in cognition, we found that the SCD-plus score was still a significant predictor, explaining more variance than objective memory performance (as seen by the contribution to *R*^2^ in the prediction model) in CSF-Aß42 and CSF Aß-42/tau ratio (Table 4).

**Table 4:** Linear regression with the number of fulfilled SCD-plus features and the number of reported SCD domains predicting AD biomarkers

Predictor variables	Dependent variables											
	CSF Aß-42-level (pg/ml)			CSF Aß-42/tau-ratio			CSF-p-tau-181 (pg/ml)			CSF total Tau (pg/ml)		
	*B*	*SE (B)*	*ß*	*B*	*SE (B)*	*ß*	*B*	*SE (B)*	*ß*	*B*	*SE (B)*	*ß*
Unadjusted model												
SCD-plus features	*R*^2^ = .054, *F* for change in *R*^2^ = 11.49***			*R*^2^ = .047, *F* for change in *R*^2^ = 10.024***			*R*^2^ = .006. *F* for change in *R*^2^ = 1.139			*R*^2^ = .001. *F* for change in *R*^2^ = .001		
	− 0.059	0.017	− 0.233***	− 0.056	0.019	− 0.217**	− .006	0.016	− 0.028	2.798E−05	0.018	0.000
SCD-I domains	*R*^2^ = .044, *F* for change in *R*^2^ = 9.213**			*R*^2^ = .029, *F* for change in *R*^2^ = 6.015*			*R*^2^ = .006, *F* for change in *R*^2^ = 1.139			*R*^2^ = .003. *F* for change in *R*^2^ = .610		
	− .062	0.020	− 0.209**	− 0.056	0.023	− 0.170*	− 0.020	0.019	− 0.077	− .016	.020	− .055
Covariate-adjusted model (age, sex, education)												
SCD-plus features	*R*^2^ = .076, *F* for change in *R*^2^ = 1.57			*R*^2^ = .110, *F* for change in *R*^2^ = 4.679**			*R*^2^ = .067, *F* for change in *R*^2^ = 4.488**			*R*^2^ = .091. *F* for change in *R*^2^ = 6.548***		
	− 0.057	0.018	− 0.225***	− 0.053	0.022	− 0.189**	− .016	0.016	− 0.074	− 0.011	0.017	− 0.043
SCD-I domains	*R*^2^ = .067, *F* for change in *R*^2^ = 1.64			*R*^2^ = .096, *F* for change in *R*^2^ = 4.953**			*R*^2^ = .075, *F* for change in *R*^2^ = 4.731**			*R*^2^ = .097. *F* for change in *R*^2^ = 6.816***		
	− 0.060	0.020	− 0.203**	− 0.048	0.022	− 0.146*	− 0.030	0.018	− 0.118	− 0.026	0.020	− 0.090
Covariate-adjusted model (age, sex, education, and delayed recall)												
SCD-plus features	*R*^2^ = .109, *F* for change in *R*^2^ = 7.35**			*R*^2^ = .135, *F* for change in *R*^2^ = 5.657*			*R*^*2*^ = .088, *F* for change in *R*^2^ = 4.298*			*R*^2^ = .115. *F* for change in *R*^2^ = 5.430*		
	− 0.053	0.017	− 0.210**	− 0.050	0.019	− 0.177**	− 1.037	0.863	− 0.083	− 0.014	0.017	− 0.056
Delayed recall	0.048	0.018	0.194**	0.046	0.019	0.167*	− 1.964	.895	− 0.152*	− 0.041	0.017	− 0.166*
	*R*^2^ = .103, *F* for change in *R*^2^ = 7.908**			*R*^2^ = .123, *F* for change in *R*^2^ = 6.040*			*R*^2^ = .095, *F* for change in *R*^2^ = 4.257*			*R*^2^ = .122. *F* for change in *R*^2^ = 5.524*		
SCD-I domains	− 0.057	0.020	− 0.193**	− 0.045	0.022	− 0.138*	− 0.031	0.018	− 0.122	− 0.028	0.020	− 0.098
Delayed recall	0.050	0.018	0.201**	0.048	0.020	0.174*	− 0.033	0.016	− 0.151*	− 0.041	0.017	− 0.167*

Full models are shown in the Additional file 1: Table S1 and Table S2

*SE* standard error, *B* unstandardized beta, *ß* standardized beta

*p < .05; **p < .01; ***p < .001

We further observed that participants endorsing a *decline in memory or language* had significantly lower age-adjusted Aß-42 levels than those who did not report a decline in these domains (Table 3). Interestingly, a reported decline in the other domains (which occurred less often than a reported decline in memory and language) was not significantly associated with Aß-42.

**Fig. 3:**
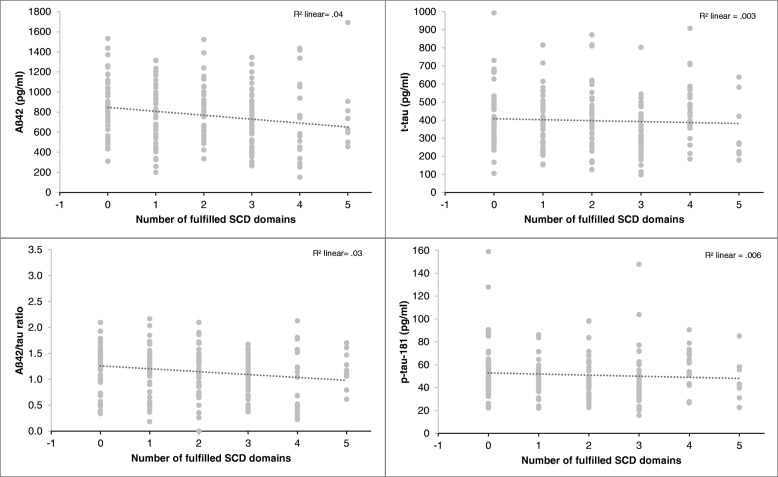
Relationship between CSF-biomarkers and the number of fulfilled SCD domains in cognitively normal individuals (*n* = 205)

The *number of reported domains* with experienced decline was also a significant predictor of lower CSF-Aß42 level (*ß* = − .209, *p* < .01) and lower CSF Aß42/tau-ratio (*ß* = − .146, *p* < .05) after including age, sex, education, and the delayed recall score to the model. For CSF-p-tau18 and total Tau, only age (total Tau *ß* = .260, *p* < .001; p-tau: *ß* = .215, *p* < .01) and delayed recall score (total tau: *ß* = − .167, *p* < .05; p-tau: *ß* = − .151, *p* < .05) were significant predictors (Table 4, Fig. 3).

## Discussion

In the present study, we demonstrate the feasibility and validity of a short semi-structured interview (SCD-I) designed to capture important aspects of SCD in the context of preclinical AD. In particular, the SCD-I captures all current experiential SCD-plus criteria within a single instrument. We here used the SCD-I to explore the quantitative and qualitative diversity of subjective cognitive decline in cognitively normal subjects at clinical or familial risk for AD, and in cognitively normal controls screened for the absence of either risk. We also established the association of SCD-plus items and of two quantitative SCD-I scores with CSF biomarkers.

### Prevalence of SCD domains and SCD-plus features

Across all three groups of these cognitively normal older adults, *memory* and *language* complaints were most frequent while complaints in the domain *planning* were relatively rare with only 10% of participants reporting them. As expected, due to the inclusion criteria, almost all SCD patients reported a decline in one or more cognitive domains within the last years. However, two thirds of the AD relatives and about half of the HC group also endorsed at least some cognitive decline. The latter is in line with community studies reporting prevalence rates from 25 to 50% of memory complaints increasing with age [29]. This indicates that SCD can be caused by other non-AD etiologies including personality traits [30], physiological aging, or the research setting [31], highlighting the need to further investigate the features characterizing SCD in the context of preclinical AD.

A *worrisome cognitive decline* was reported by most of the SCD subjects, again, an expected finding given that a worrisome decline reported during the memory clinic screening was required for inclusion. Interestingly, 29.2% of the AD relatives and even 14.5% of the HC group reported at least one worrisome cognitive decline during the baseline SCD interview. The latter finding was contrary to our expectations since these “control” individuals had negated a question regarding any worrisome self-perceived cognitive decline during the initial telephone screening. Yet, they expressed some concern to the clinician during the SCD interview. In contrast, 10% of the SCD patient group did *not* report worries in the SCD-I although expression of concerns regarding the self-perceived cognitive decline to the physician of the memory clinic at screening was a mandatory inclusion criterion. These discrepancies may be due to several reasons, including temporal instability of measurements and subjective reports in general, the difference between settings, and possibly an undeclared interest of some healthy volunteers to participate in a study which they imagine to confer some health benefit [32]. Consistency of worries over time has been shown to relate to clinical progression [6] and thus will be an interesting issue for future analyses of longitudinal SCD-I data.

Most SCD participants (81%) reported an onset of a decline *within the last 5 years.* This is perfectly in line with the reported onset in the SCIENCE SCD-cohort, where 83% reported an onset within the last 5 years [33]. Interestingly, those HC and AD relatives who reported any decline frequently indicated a more distant onset. This suggests a different pattern of perceived onset of decline in cognitively unimpaired memory clinic patients.

The SCD-plus feature *performing subjectively worse than others* was reported least frequently, endorsed by 30% of the SCD patients but only by 1% in the healthy control. Finally, the *confirmation of any complaints by an informant* occurred for the majority of SCD participants (58%). Interestingly, 38% of informants of those controls who reported any decline also confirmed an observed decline in at least one domain, as did 44% of the informants of AD relatives endorsing any cognitive decline. Thus, there is a considerable overlap between groups not only regarding any self-reported decline, but also regarding the degree of confirmation by the informants.

### Relation of SCD-I items and SCD-I scores with AD CSF biomarkers

In line with the first interim report from the DELCODE study based on a smaller sample [28], we found that SCD participants had lower age-adjusted CSF-Aß42 levels and lower CSF-Aß42/Tau ratios than HC, while the total Tau level and the p-Tau-181 level did not differ between groups.

The SCD-plus sum score and the SCD-I domain score were significantly and specifically associated with measures of amyloid pathology, and to the same extent with a derived amyloid/Tau ratio, but not with p-Tau181 or t-tau level alone. The associations with amyloid are in line with previous studies using either CSF amyloid measures [14, 25] or amyloid PET [16, 34]. Like in the present study, CSF-Tau was not associated with quantitative SCD in the studies of [14, 25]. However, significantly positive associations between quantitative SCD and regional Tau measured with flortaucipir PET have been reported [16, 35]. This discrepancy may be due to the low correlation between CSF Tau and flortaucipir tracer uptake in early disease stages [36].

Three of the suggested SCD-plus features were significantly associated with amyloid pathology: *experienced decline in memory*, *onset of subjective decline within the last 5 years*, and *confirmation by an informant.* The association with worries was almost significant (*p* = .056), which bears mentioning because a recent study with cognitively normal memory clinic patients [33] and a community-based study [20, 37] also reported an association of worries with amyloid pathology. To our knowledge, only the Amsterdam SCIENCe cohort has tested associations of all SCD-plus criteria with amyloid pathology, using different questionnaires plus some questions similar to those of the SCD-I interview to reflect the criteria. Two studies from this cohort [20, 33], in contrast to our own study, did not find associations with “decline in memory” and “onset within 5 years”. Apart from differences in assessment, this may be due to the younger age of the SCIENCe cohort as compared to the DELCODE cohort (64 versus 69 years on average). However, an association of subjective memory decline (e.g., [7]) and cognitive ratings by informants [38] with amyloid pathology has been found before. Our study appears to be the first one directly testing and validating the SCD-plus criterion “onset within the last 5 years”.

The biomarker associations of the single SCD domains revealed that perceived decline in the memory and language domain showed the highest associations with AD biomarkers, which is in line with studies suggesting that memory complaints are the best predictor of incident MCI [37] or that memory-related complaints are associated with PIB retention in healthy older adults [7]. However, to our knowledge, this is the first study reporting an association of Aß42 with subjectively experienced decline in the domain of language abilities. This pattern is consistent with the earliest neuropsychological deficits in AD starting with decline in episodic memory followed by deficits in language [39].

We also observed a relationship between objective memory performance and CSF-Aß42 level irrespective of the subjective complaints. While subtle objective cognitive decline can be expected in “late” preclinical AD [40], we showed that subjective cognitive decline is equally and independently predictive of amyloid abnormality in cognitively normal individuals. This extends findings of a previous study which also found an independent association of subjective and objective cognitive performance with CSF-Aß in patients with MCI [25].

### Strengths and limitations

The current brief SCD interview has been derived from the assessment routine in memory clinics and standardizes the assessment of those SCD features which are currently considered relevant for assessing suspected preclinical and prodromal AD. Aside from establishing the presence or absence of each of these features, it offers summary measures of quantitative SCD, which in the current study predicted the presence of amyloid pathology.

The SCD-I is a direct operationalization of the SCD-plus criteria and therefore has high content validity. While this is not the only conceivable operationalization, it is one which is frequently used in clinical assessment, e.g., in DSM-based interviews, like the SCID [41], or in questionnaires directly based on diagnostic criteria (like the PHQ-9 [42]). The SCD-I discriminated well between the HC and SCD groups in our study. This is somewhat circular for the items of cognitive decline and related concerns, as these SCD-plus criteria were used for group definition at inclusion. However, also the other SCD-plus items assessed at baseline with the SCD-I markedly differ between the groups. Furthermore, most SCD-I items, and both SCD-I summary scores, were associated with CSF-amyloid, implicating that it captures, to some degree, AD-related cognitive concerns. In sum, this provides a first validation of the SCD-I as a measure for SCD. This does not imply that this assessment method is superior to others, e.g., questionnaire-based methods. For example, the SCD-Q [43] captures many of the SCD-plus items (it lacks the question of comparison with others of the same age, and asks for perceived decline in the last 2 years, rather than 5 years). In an elderly population sample enriched for family history of AD, larger SCD-Q scores were associated with objective cognitive impairment and confirmation of decline by an informant predicted cerebral volume reduction in AD-related brain areas [44]. More data are needed to compare the prediction of the same outcomes by different SCD assessment methods.

One limitation of the present SCD-I is that it directly asks for an experienced or observed decline in five neuropsychological domains, using global and commonly used terms like memory, language, or planning. Whether subjects “correctly” identify their specific problems as being related to one of those domains is unknown. However, subjects can endorse deficits in many domains as opposed to only one or two, and the domain score, like the SCD-plus score, seems to capture SCD severity, as it is related to AD pathology. The SCD domain scores can be calculated for reports of patients and informants alike, so that the difference between both scores could be used to examine the shift from a hyperawareness to hypoawareness of cognitive deficits with the progression of AD [45].

Current research suggests that other specific aspects or higher-order thinking, e.g., self-reports of confusion, are also related to AD pathology in cognitively normal individuals [15]. To identify alternative descriptions of experienced and possibly pathological cognitive change, we have added an open initial question in the SCD-I asking for *any* observed cognitive change during recent years. The recorded answers will be analyzed with the help of qualitative methods [46, 47] and may give rise to the identification of new AD-related SCD features not captured by this first iteration of the SCD-I.

Furthermore, it should be noted that individuals included in the present study were recruited in the memory clinic (SCD patients) as well as from the community (HC and relatives). Evidence suggests that the active process of seeking medical help due to self-perceived cognitive decline is a factor with potential prognostic value for the presence of AD pathology [12, 13]. Validation of the SCD-I in other samples will be another research goal of the future.

## Conclusion

Findings support the use of interview-based approaches for the assessment of AD-related subjective cognitive decline. In this study, detailed questions on perceived decline in different cognitive domain and on the presence/absence of the SCD-plus features were related to AD biomarkers in cognitively normal participants of the DELCODE study. Combining information on perceived decline in multiple cognitive domains and SCD-plus features is useful for prediction of underlying AD pathological change in these individuals. The consistent report of worries/non-worries is possibly an additional SCD-plus feature to be considered in future SCD studies.

## Additional file

Additional file 1: Subjective Cognitive Decline Interview (SCD-I). **Table S1.** Linear regression with the number of fulfilled SCD-plus features predicting AD biomarkers. **Table S2.** Linear regression with the number of reported SCD-I domains predicting AD biomarkers. (PDF 137 kb)

## Data Availability

The data, which support this study, are not publically available, but may be provided upon reasonable request.

## Abbreviations

AD: Alzheimer’s disease
ANOVA: Analysis of variance
APOE: Apolipoprotein E
Aß: ß-Amyloid
CDR: Clinical Dementia Rating
CDR-SOB: Clinical Dementia Rating-sum of boxes
CERAD: Consortium to Establish a Registry for Alzheimer’s Disease
CSF: Cerebrospinal fluid
DELCODE: DZNE-Longitudinal Cognitive Impairment and Dementia Study
DZNE: German Centre for Neurodegenerative Diseases (Deutsches Zentrum für Neurodegenerative Erkrankungen)
eCRF: Electronic case report form
FBB: 18F-Florbetaben
FDG: 18F-Fluordesoxyglucose
fMRI: Functional magnetic resonance imaging
GDS: Geriatric Depression Scale
HC: Healthy controls
ICD-10: International Classification of Diseases
MAC-Q: Memory Assessment Clinic-Questionnaire
MCI: Mild cognitive impairment
MMSE: Mini-Mental State Examination
MRI: Magnetic resonance imaging
p-Tau-181: Tau phosphorylated at position 181
SCD: Subjective cognitive decline
SCD-I: Subjective cognitive decline interview
SD: Standard deviation
SOP: Standard operation procedure
SPSS-23: Statistical Package for the Social Sciences, 23rd Edition
