# Supplementary material for: Which features of subjective cognitive decline are related to amyloid pathology? Findings from the DELCODE study
Source: Alzheimers Res Ther. 2019 Jul 31;11:66. doi: 10.1186/s13195-019-0515-y (PMC6668160; doi:10.1186/s13195-019-0515-y)
Supplement: Supplementary file 1 — Subjective Cognitive Decline Interview (SCD-I). Table S1. Linear regression with the number of fulfilled SCD-plus features predicting AD biomarkers. Table S2. Linear regression with the number of reported SCD-I domains predicting AD biomarkers. (PDF 137 kb) [file 13195_2019_515_MOESM1_ESM.pdf]

Miebach et al.

Appendix. **Subjective Cognitive Decline Interview 1.0**

(as used in the DELCODE study. Jessen et al. 2018)

1. Open question

The purpose of this question is to elicit a short free report of the most salient cognitive changes experienced during recent years.

The interviewer may take notes and may classify the presented complaints into one or more of the following categories: memory. language. organization and planning. attention and concentration. other.

Qualitative content analysis can be applied to recorded interviews.

(Responses to this open question were not analysed in the present paper)

„During the past few years. have you noticed any changes in your mental abilities? Could you give an example from everyday life?“

2. Structured interview with participant/patient

Five questions on experienced cognitive decline are asked by the interviewer. Only for the endorsed items. the interviewer asks up to five additional questions A to E. as stated below.

"Now I will ask you about specific changes of mental abilities which you may have observed"

**1.**	***"Has your memory become worse?"***	☐ yes	☐ no
	A. ☐ B. ☐ C. ☐ D. ☐ E. ☐☐ months ago		
**2.**	***"Do you have increasing word finding difficulties?"***	☐ yes	☐ no
	A. ☐ B. ☐ C. ☐ D. ☐ E. ☐☐ months ago		
**3.**	***"Do you have increasing difficulties planning ahead or staying organized?"***	☐ yes	☐ no
	A. ☐ B. ☐ C. ☐ D. ☐ E. ☐☐ months ago		
**4.**	***"Do you make increasingly more errors when not paying full attention to a task?"***	☐ yes	☐ no
	A. ☐ B. ☐ C. ☐ D. ☐ E. ☐☐ months ago		
**5.**	***"Have you noticed any other cognitive changes?***<br>***Please describe briefly:***<br>____	☐ yes	☐ no
	A. ☐ B. ☐ C. ☐ D. ☐ E. ☐☐ months ago		

Coding:

A. Does this worry you?

0 = no. 1 = yes

B. When was the onset of this decline?

1 = within the last six months. 2 = between six months and two years ago. 3 = between two and five years ago. 4 = more than five years ago

C. Do you think your performance in this area is worse as compared to other people of your age?

0 = no. 1 = yes

D. Have you talked to a physician about these problems?

0 = no. 1 = yes. if yes. please anwer

E. when for the first time?

3. Structured interview with an informant

Six questions on observed cognitive decline are asked by the interviewer. Only for the endorsed items. the interviewer asks about the onset of the change.

"Now I will ask you about specific changes of mental abilities which you may have observed in the (participant/patient)"

**1.**	***"Has her/his memory become worse?"***<br>A. ☐	☐ yes	☐ no
**2.**	***"Does (s)he have increasing word finding difficulties?"***<br>A. ☐	☐ yes	☐ no
**3.**	***"Does (s)he have increasing difficulties planning ahead or staying organized?"***<br>A. ☐	☐ yes	☐ no
**4.**	***"Does (s)he make increasingly more errors when not paying full attention to a task?"***<br>A. ☐	☐ yes	☐ no
**5.**	***"Have you noticed any other cognitive changes?***<br>***Please describe briefly"***<br>____	☐ yes	☐ no
**6.**	***"Have you noticed any changes in behavior or personality?"***<br>A. ☐	☐ yes	☐ no

Coding:

A. When was the onset of this decline?

1 = within the last six months. 2 = between six months and two years ago. 3 = between two and five years ago. 4 = more than five years ago

Appendix. Table 1. Linear regression with the number of fulfilled SCD-plus features predicting AD-biomarkers

Predictor variables	Dependent variables											
	**CSF Aß-42-level (pg/ml)**			**CSF Aß-42/tau-ratio**			**CSF- total Tau (pg/ml)**			**CSF-p-tau-181 (pg/ml)**		
	B	*SE (B)*	*ß*	*B*	*SE (B)*	*ß*	*B*	*SE (B)*	*ß*	*B*	*SE (B)*	*ß*
Unadjusted model												
	R^2=.054. F for change in R^2 =**11.49*****			R^2=.047. F for change in R^2 =**10.024*****			R^2=.001. F for change in R^2 =.001			R^2=.001. F for change in R^2 =.150		
Sum of fulfilled SCD-plus features	-0.059	0.017	**-0.233*****	-0.056	0.019	-0.056	2.798E-05	0.018	0.000	-.006	0.016	-0.028
Covariate-adjusted model												
	R^2=.076. F for change in R^2= 1.57			R^2=.110. F for change in R^2=**4.679****			R^2=.091. F for change in R^2=**6.548*****			R^2=.067. F for change in R^2=**4.488****		
Sum of fulfilled SCD-plus features	-0.057	0.018	**-0.225*****	-0.053	0.022	**-0.189****	-0.011	0.017	-0.043	-.016	0.016	-0.074
Age	-0.004	0.006	-0.056	-0.017	0.006	**-0.186****	0.020	0.005	**0.258*****	0.015	0.005	**0.214****
Education	0.014	0.011	0.095	0.013	0.012	0.073	-0.001	0.011	-0.007	0.003	0.010	0.025
sex	0.114	0.064	0.131	0.153	0.069	**0.158***	-0.117	0.062	-0.137	-.094	0.057	-0.124
	R^2=.109. F for change in R^2= **7.35****			R^2=.135. F for change in R^2=**5.657***			R^2=.115. F for change in R^2=**5.430***			R^2=.088. F for change in R^2=**4.298***		
Sum of fulfilled SCD-plus features	-0.053	0.017	**-0.210****	-0.050	0.019	**-0.177****	-0.014	0.017	-0.056	-1.037	0.863	-0.083
Age	-0.002	0.006	-0.025	-0.015	0.006	**-0.163***	0.019	0.005	**0.234*****	0.677	0.276	**0.196****
Education	0.009	0.011	0.063	0.009	0.012	0.052	0.003	0.011	0.020	0.414	0.541	0.051
sex	0.067	0.065	0.077	0.106	0.071	0.110	-0.077	0.064	-0.090	-3.539	3.225	-0.080
Delayed recall	0.048	0.018	**0.194****	0.046	0.019	0.167*	-0.041	0.017	**-0.166***	-1.964	.895	**-0.152***

Notes. *p<.05; **p<.01***p<.001; *SE*= Standard Error

Appendix . Table 2. Linear regression with the number of reported SCD-I-Domains predicting AD-biomarkers

Predictor variables	Dependent variables											
	**CSF Aß-42-level (pg/ml)**			**CSF Aß-42/tau-ratio**			**CSF- total Tau (pg/ml)**			**CSF-p-tau-181 (pg/ml)**		
	B	*SE (B)*	*ß*	*B*	*SE (B)*	*ß*	*B*	*SE (B)*	*ß*	*B*	*SE (B)*	*ß*
Unadjusted model												
	R^2=.044. F for change in R^2 =9.213**			R^2=.029. F for change in R^2 =6.015*			R^2=.003. F for change in R^2 =.610			R^2=.006. F for change in R^2 =1.139		
SCD-I Domains	-.062	0.020	-0.209**	-0.056	0.023	-0.170*	-0.016	0.020	-0.055	-0.020	0.019	-0.077
Covariate-adjusted model												
	R^2=.067. F for change in R^2= 1.64			R^2=.096. F for change in R^2=4.953**			R^2=.097. F for change in R^2=6.816***			R^2=.075. F for change in R^2=4.731**		
SCD-I Domains	-0.060	0.020	-0.203**	-0.048	0.022	-0.146*	-0.026	0.020	-0.090	-0.030	0.018	-0.118
Age	-0.005	0.006	-0.063	-0.018	0.006	-0.197**	0.021	0.005	0.260***	0.015	0.005	0.215**
Education	0.013	0.011	0.091	0.012	0.012	0.073	0.000	0.011	-0.001	0.004	0.010	0.031
sex	0.115	0.064	0.132	0.154	0.070	0.159*	-0.119	0.062	-0.140	-0.097	0.057	-0.127
	R^2=.103. F for change in R^2= 7.908**			R^2=.123. F for change in R^2= 6.040*			R^2=.122. F for change in R^2= 5.524*			R^2=.095. F for change in R^2=4.257*		
SCD-I Domains	-0.057	0.020	-0.193**	-0.045	0.022	-0.138*	-0.028	0.020	-0.098	-0.031	0.018	-0.122
Age	-0.003	0.006	-0.033	-0.015	0.006	-0.172*	0.019	0.005	0.235***	0.014	0.005	0.197**
Education	0.009	0.011	0.060	0.008	0.012	0.047	0.004	0.011	0.025	0.007	0.010	0.056
sex	0.065	0.066	0.075	0.105	0.072	0.109	-0.079	0.064	-0.092	-0.064	0.059	-0.084
Delayed recall	0.050	0.018	0.201**	0.048	0.020	0.174*	-0.041	0.017	-0.167*	-0.033	0.016	-0.151*

Notes. *p<.05; **p<.01***p<.001; *SE*= Standard Error
